# Diagnostics for Developing Countries

**DOI:** 10.3390/diagnostics5020200

**Published:** 2015-05-19

**Authors:** Ruth McNerney

**Affiliations:** TB Alert, Community Base, 113 Queens Road, Brighton, BN1 3XG, UK; E-Mail: Ruth.Mcnerney@gmail.com; Tel.: +44-7557-020305

**Keywords:** IVD, point-of-care, tuberculosis, HIV, regulation, diagnostic test, infectious disease

## Abstract

Improving the availability of high quality diagnostic tests for infectious diseases is a global priority. Lack of access by people living in low income countries may deprive them of life saving treatment and reduces opportunities to prevent onward transmission and spread of the disease. Diagnostic laboratories are often poorly resourced in developing countries, and sparsely distributed. Improved access may be achieved by using tests that do not require laboratory support, including rapid tests for use at the point-of-care. Despite increased interest, few new *in vitro* diagnostic (IVD) products reach the majority populations in low income countries. Barriers to uptake include cost and lack of robustness, with reduced test performances due to environmental pressures such as high ambient temperatures or dust. In addition to environmental factors test developers must consider the local epidemiology. Confounding conditions such as immunosuppression or variations in antigen presentation or genotype can affect test performance. Barriers to product development include access to finance to establish manufacturing capacity and cover the costs of market entry for new devices. Costs and delays may be inflated by current regulatory preregistration processes to ensure product safety and quality, and more harmonized approaches are needed.

## 1. Introduction

Many low income countries with weak and poorly resourced health care systems are burdened with levels of infectious disease detrimental to their socio-economic development. Notwithstanding the recent Ebola pandemic, tuberculosis (TB) and HIV/AIDS remain major public health concerns and are the most frequently recorded cause of adult death in parts of sub-Saharan Africa [1]. Average life expectancies in sub-Saharan Africa have increased with the rollout of anti-retroviral treatment but remain less than sixty years of age [2,3]. Failure to control communicable diseases is in part due to the failure to correctly diagnose and treat infectious cases, when opportunities to prevent onward transmission are missed. The World Health Organization (WHO) estimates that only 64%, of new TB cases are detected and notified, suggesting over 3 million cases of this highly contagious disease are missed each year [4]. The Joint United Nations Programme on HIV/AIDS (UNAIDS) estimates that less than half of people living with HIV in sub-Saharan Africa know their status and that globally 19 million individuals remain unaware [3]. The situation for children is also critical. Without HIV treatment, it is estimated that half of children living with HIV will die by age two with peak mortality 8–12 weeks after birth [5]. In 16 countries in sub-Saharan Africa less than half the children born to HIV positive mothers are screened for the virus and in some countries less than one in 10 HIV-exposed children are tested [3].

Inadequate provision of diagnostic services for infectious diseases is a serious impediment to improving the health of a nation [6]. Laboratories in developing countries are often sparsely distributed, and access may be limited by economic or geographical factors. Where they do exist clinical laboratories are often under resourced and amenities such as electrical supply and water may be unreliable [7]. Shortage of skilled technical personnel is also a problem in some countries, particularly in rural areas. Particular diagnostic tests may not be available to the majority population due to their high cost or lack of robustness. In addition, some manufacturers may be reluctant to supply countries if return on their investment is likely to be low or where it may be difficult to establish effective mechanisms for product distribution or technical support [8]. For some tropical and neglected diseases appropriate tests have not yet been developed [9]. Weak regulation has also contributed to the sub-optimal provision of diagnostic services and in some countries tests of unknown or dubious quality are sold without hindrance [10,11].

Technological advances and the development of novel devices that can be used outside of the laboratory have the potential to overcome some of the challenges faced by healthcare providers in developing countries [12]. Rapid tests for use at the point-of-care offer new solutions for detecting communicable disease [13]. However, despite increased interest in academic and other circles, few new tests suitable for use in low income countries reach the market place. This article discusses factors influencing the development of *in vitro* diagnostic devices for developing countries with special consideration of the major infectious diseases, TB and HIV/AIDS.

## 2. The Diagnostics Pipeline

**Figure 1:**
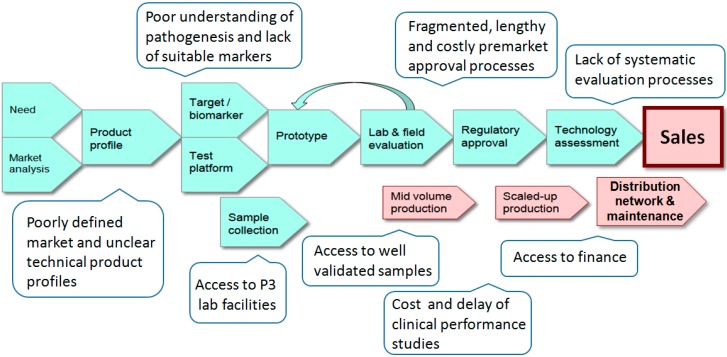
The *in vitro* diagnostic (IVD) pipeline and obstacles encountered when developing tests for infectious disease in developing countries.

The development, production and marketing of a new *in vitro* diagnostic device is a complex undertaking, made more challenging if it is intended for use in developing countries. As demonstrated in Figure 1 there are hurdles at all stages of the R&D pipeline. Although the clinical and humanitarian justification for a new test may be obvious there is often little guidance for test developers regarding the product profile or technical specification. To aid developers of tests for HIV/AIDS a series of consensus target product profiles (HIV detection, early infant diagnosis, viral load and CD4 counts) have been created by a panel of international experts [14]. Similarly, lists for priority products for TB have also been published [15,16]. These lists are not prescriptive and a test that does not entirely meet the suggested requirements may still be of value. For example, a test that has less than ideal sensitivity but that is stable at elevated temperatures could prove extremely beneficial in tropical settings. It should also be noted that affordability may out-way the need for sensitivity in some countries and that the acceptable specificity of a test will vary according to the local incidence of the condition.

Whereas funding may be available for academic research towards identifying novel biomarkers, funds to establish manufacturing capacity or networks for distribution and maintenance are often more difficult to source. Poorly defined markets and low expectation of return on investment impede access to finance from commercial sources and public or philanthropic funding may be required [8].

## 3. Product Profiles

### 3.1. Technological Considerations

It is not uncommon for devices that perform well in a controlled environment to fail when used in tropical regions and one of the greatest challenges facing test developers is to manufacture tests able to withstand the environmental and climatic demands of poorly resourced settings. Optimization studies using prototype devices at sites of intended use should be undertaken early in product development. Exposure to extremes of temperature during transport and storage is a particular problem [17] and stability studies should be carried out at evaluated temperatures to establish shelf life. In addition to damaging instrumentation, dust can also adversely affect test performance and false positive results due to dust and environmental contamination have recently been reported for an immunoassay to detect lipoarabinomannan, a mycobacterial cell wall antigen indicative of TB disease [18]. Improvements in infrastructure such as instillation of dust filters, air conditioning or maintaining a delivery cold chain will inflate the costs of devices, reducing affordability. An example of this is the GeneXpert analyzer (Cepheid Inc. Sunnyvale, CA, USA) an easy-to-use platform for nucleic acid amplification that was developed for homeland security use in the USA. Adaption of the technology to detect *Mycobacterium tuberculosis* in sputum suggested high sensitivity and specificity could be achieved [19]. However, following rollout of the test to TB endemic countries reports have emerged of high failure rates in tropical countries [20,21]. The study authors proposed erratic power supplies, high ambient temperatures and dust to have contributed to the high failure rate. The necessity to improve the infrastructure of some laboratories to prepare them for GeneXpert increases set-up costs, which in a Nigerian study were reported as between USD 2622 and USD 9716 per laboratory [22]. Ideally an IVD for developing countries should be heat stable, resistant to dust and independent of mains electricity. The test should also be easy to perform with a minimum of training and provide a result in time to influence decisions regarding management of the patient. Portability is also a useful asset, as it would allow the device to be used either at the point-of-care, or as screening device within the community thus increasing access for populations in remote settings. A further consideration is disposal of the devices, as facilities for safe removal of used tests may not be available.

The ideal diagnostic test would have an accuracy of 100% but such perfection is not achievable in routine clinical practice and compromises may be needed between accuracy and accessibility. A less sensitive rapid test for use in the community may detect more cases than a highly sensitive test that can only be used in a specialized laboratory [23]. The specificity required of the test will differ according to its intended use as tests to direct treatment demand a higher specificity than those used for screening, where follow up confirmatory tests are performed. It also should be noted that the predictive value of a test is dependent on the prevalence of the condition in the population and devices intended for settings with a low prevalence require very high specificities to minimize the incidence of false positive test results [24].

### 3.2. Epidemiological Considerations

Patterns of disease in developing countries often differ from those of richer countries and when designing a new test, developers must take into account the local epidemiology at the site of intended use. Test design must accommodate evolutionary differences in pathogens that lead to geographic variation in antigen presentation or nucleic acid signals. Confounding conditions can alter test performance, decreasing, or occasionally increasing, sensitivity and specificity. The deleterious effect of HIV driven immunosuppression on tests to detect host response signals is well known [25,26]. Co-infection with HIV also depresses the sensitivity of smear microscopy and sputum based nucleic acid tests for detecting pulmonary TB [19,27]. Conversely, tests for the TB antigen lipoarabinomannan in urine increase in sensitivity with patients with CD4 low counts [28]. The predictive value of HIV test results may be reduced by tropical diseases such as schistosomiasis or malaria and elevated levels of rheumatoid factor which have all been associated with false positive immunoassay results [29,30].

## 4. Logistical Challenges

Variation in the biology and pathogenesis of infectious diseases necessitates multiple approaches to their detection. For pathogens such as malaria or HIV that circulate in bodily fluids, or those that elicit a characteristic immune response the selection of samples to test is straight forward. For diseases such as TB or leishmaniasis sampling is more problematic as direct detection of the organisms is often difficult, particularly during early stages of the disease when treatment interventions that could prevent further transmission are most effective [9]. Unfortunately tests for immune response to these diseases often lack specificity and cannot differentiate past or latent infection from active disease [12]. One of the challenges shared by all test developers is access to well characterized samples with which to validate their tests. To address this issue the WHO and some not for profit organisations have established specimen banks where clinical samples are curated and made available to test developers designing tests for developing countries. An example is the tuberculosis specimen bank [31] that is currently managed by the Foundation for Innovative New Diagnostics (FIND) which may be accessed via the website www.finddiagnostics.org/programs/tb/find_activities/tb_specimen_bank. Characterization of samples from TB patients is particularly problematic. Extra-pulmonary disease and pediatric cases are often difficult to detect and evidence from autopsy studies from settings with a high prevalence of TB suggests undiagnosed extra pulmonary disease may be present in a proportion of the population [32,33]. Unlike sputum based tests, those that use blood or urine may detect all forms of the disease and misclassification of samples used in validation studies due to a failure to detect extra-pulmonary disease may lead to inaccurate estimates of test specificity.

There are additional challenges when working with infectious diseases, particularly deadly airborne pathogens such as *M. tuberculosis* which require microbiological safety level III (P3) [34]. The need for specialist handling and infection control facilities increases costs and is a disincentive to working with hazardous pathogens. Once validated in laboratory studies the performance of the device must be determined at sites of intended use. For devices intended for use at the point-of-care studies should take place in the clinic as studies undertaken in a referral or research laboratory may give a false impression of the robustness of the device.

Quality assurance and maintenance of equipment is often difficult to achieve in developing countries, particularly for devices used outside of the laboratory network and at the point-of-care. This may easier for devices that incorporate wireless or mobile phone technology, where information on usage and performance of the device can be collected remotely. However, connectivity (the state of being connected), is in its infancy for *in vitro* diagnostics and international standards or guidelines have not been implemented.

## 5. IVD Regulation in Developing Countries

Registration and regulation of *in vitro* diagnostic devices is frequently a complex and expensive process that delays market entry [10]. National Regulatory Authorities (NRA) are mandated to ensure the safety of the populations they serve, but in many countries processes to regulate IVDs are not well developed [35,36]. In the absence of regulatory control by the NRA some national disease control programs or research laboratories undertake their own studies on new diagnostic tests. Examples of non-regulatory agencies that undertake product evaluations for priority diseases are the National Health Laboratory Service (NHLS) of South Africa and the Kenya Medical Research Institute (KEMRI). Clinical performance studies to assess the sensitivity and specificity of a test can delay access to the device for years. Redundant duplication, where additional studies are requested that offer little additional scientific benefit increase costs unnecessarily, making the final product less affordable [12]. The first point-of-care test to enumerate CD4 cells to be widely marketed has been reported to have undergone over 60 evaluation studies. Recognition of these barriers to international trade and the need to reduce unnecessary duplication has led to a series of initiatives towards more harmonized regulation using standardized procedures. Global and regional organizations such as the International Medical Devices Regulatory Forum, and the Asian and the Pan African Harmonization Working Parties have been established to promote more efficient regulatory practices.

A new iniative designed to accelerate access to new point-of-care diagnostic technologies for persons living with HIV has been launched with funding from UNITAID. A consortium of not-for-profit organizations has established a pilot study aimed at improving the efficiency of gathering data on the clinical performance of tests for viral load, early infant diagnosis and CD4 counts [12]. Standardized study protocols have been developed by an international group of experts and made available online [37]. Following an open call for expressions of interest a network of African study sites has been established [38]. Manufacturers seeking evaluation of their test may commission studies at individual sites of their choice. Independent monitoring of compliance with the agreed protocols will ensure the quality and independence of studies. Data generated will be jointly reviewed by a panel of international experts and representatives from NRA. Manufacturers, or their distributors, will then be free to submit the data to support premarket regulatory approval. It is hoped that this streamlined process will lead to a reduction in the number of studies requested by individual countries. In a separate initiative, Partner States of the East African Community, namely the Republics of Burundi, Kenya, Rwanda, Uganda and the United Republic of Tanzania (mainland Tanzania and Zanzibar), have agreed to harmonize regulation of medical devices, including IVDS. In doing so, they will be the second economic community to do so, following countries of the European Union.

### Prequalification

Low income countries receive donor support for the procurement of IVD for priority diseases such as HIV/AIDS, malaria and TB. Donors face a dilemma when selecting products that do not have regulatory approval as they do not have independent guidance on their safety or the quality of manufacture. Mechanisms to assist donor agencies include advice from agencies such as the US Centers for Disease Control and Prevention. WHO has a program to examine the quality and safety of tests for HIV and malaria in a process called prequalification. Advice to manufacturers regarding the process and a list of successfully prequalified IVD is available on their website [39]. WHO does not have a legal mandate to regulate medical products but prequalification is a helpful guide where recommendations from NRA are not yet available. TB tests have not so far been included in the prequalification program. The WHO TB Strategic and Technical Advisory Group (STAG TB) have endorsed some technologies following review of data on their performance published in the scientific press and provided by test developers. They have adopted the GRADE system of evaluating evidence where the strength of recommendations may be moderated dependent on the quality of the evidence that is available for review [40,41]. However, systematic review of product safety and quality of manufacture is not undertaken.

## 6. Conclusions

Control of infectious disease in developing countries is hindered by the suboptimal provision of diagnostic services and action is needed to remedy the situation. Encouragement is needed to promote innovation to address the needs of populations that are blighted by diseases such as HIV/AIDS and TB. Laboratories are often inaccessible, poorly resourced and lacking basic amenities and new tests are needed that can be used outside of the laboratory offering improved access to diagnosis, particularly for persons living in remote or rural areas. Recent advances in technologies for sample manipulation and detection offer new possibilities to develop easy to use, rapid tests. Such tests should be robust and capable of withstanding the temperatures and dust levels found in tropical climates. They also have to be affordable. Despite the considerable hurdles facing test developers and manufacturers, new devices continue to be developed; however, many promising technologies fall by the wayside and do not enter the market. Efforts to streamline regulatory processes should be encouraged to reduce unnecessary costs and accelerate premarket approval of devices found safe and beneficial the population.
